# Apex1 safeguards genomic stability to ensure a cytopathic T cell fate in autoimmune disease models

**DOI:** 10.1172/JCI183671

**Published:** 2024-12-31

**Authors:** Xiang Xiao, Yong Du, Si Sun, Xiaojun Su, Junji Xing, Guangchuan Wang, Steven M. Elzein, Dawei Zou, Laurie J. Minze, Zhuyun Mao, Rafik M. Ghobrial, Ashton A. Connor, Wenhao Chen, Zhiqiang Zhang, Xian C. Li

**Affiliations:** 1 Immunobiology and Transplant Science Center and Department of Surgery,; 2 Department of Cardiovascular Sciences, and; 3 J.C. Walter Jr. Transplant Center and Conover Center for Liver Diseases and Transplantation, Houston Methodist Hospital, Houston, Texas, USA.; 4 Department of Surgery, Weill Cornell Medical College of Cornell University, New York, New York, USA.

**Keywords:** Autoimmunity, Immunology, Autoimmune diseases, T cells

## Abstract

T cells have a remarkable capacity to clonally expand, a process that is intricately linked to their effector activities. As vigorously proliferating T cell also incur substantial DNA lesions, how the dividing T cells safeguard their genomic integrity to allow the generation of T effector cells remains largely unknown. Here we report the identification of the apurinic/apyrimidinic endonuclease-1 (Apex1) as an indispensable molecule for the induction of cytopathic T effectors in mouse models. We demonstrate that conditional deletion of *Apex1* in T cells resulted in a remarkable accumulation of baseless DNA sites in the genome of proliferating T cells, which further led to genomic instability and apoptotic cell death. Consequently, *Apex1-*deleted T cells failed to acquire any effector features after activation and failed to mediate autoimmune diseases and allergic tissue damages. Detailed mutational analyses pinpointed the importance of its endonuclease domain in the generation of T effector cells. We provide further evidence that inhibiting the base repair activities of Apex1 with chemical inhibitors similarly abrogated the induction of autoimmune diseases. Collectively, our study suggests that Apex1 serves as a gatekeeper for the generation of cytopathic T cells and that therapeutically targeting Apex1 may have important clinical implications in the treatment of autoimmune diseases.

## Abstract 2

The DNA base excision repair pathway maintains the genomic stability of activated T cells, allowing their differentiation into cytopathic T effector cells in autoimmunity.

## Graphical abstract

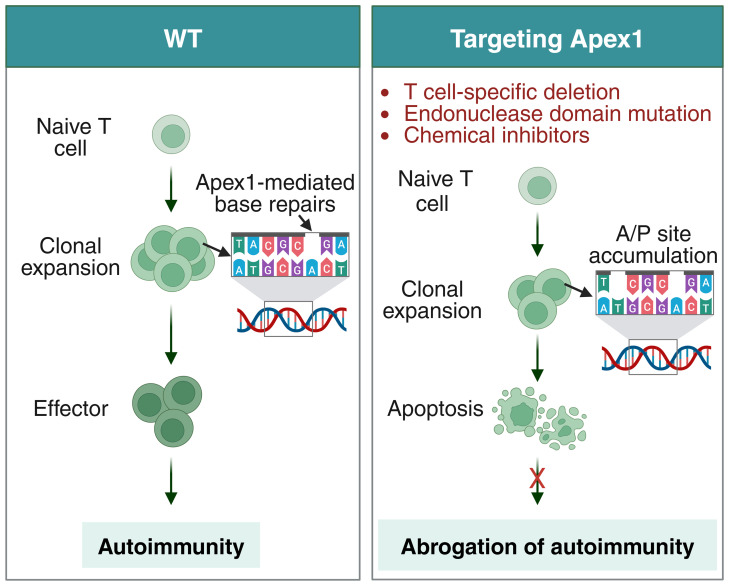

## Introduction

Autoimmune diseases are a significant issue in the clinic, affecting an estimated 50 million Americans. Thus, uncovering the fundamental mechanisms involved, along with the design of new therapeutic strategies, remains an important area of ongoing research. In essence, productive immune responses, including immune-mediated diseases, demand robust proliferation of antigen-specific T cells (1), in which vigorous DNA replication is one of the key events in propagation of daughter cells. However, DNA replication also exposes DNA bases (both purine and pyrimidine) to toxic oxidative metabolites, thus rendering them especially vulnerable to DNA base damage (2). This is mainly due to high levels of reactive oxygen species produced by the metabolically active T cells as well as the susceptibility of DNA bases to chemical modifications (3, 4). Moreover, DNA bases can also be extensively modified during spontaneous depurination upon productive T cell activation (2). Apparently, if left unrepaired, the damaged or modified DNA bases are capable of disrupting DNA base pairing (5), blocking DNA replication forks (6), and enabling DNA strand breaks and mutagenesis (2), events that may eventually lead to cellular dysfunctions or even cellular demise (7, 8).

A key question that remains to be addressed is how the highly proliferating T cells safeguard their genomic integrity to allow the generation of cytopathic T effector cells in a timely fashion. In many other cell types, especially tumor cells, the modified or damaged DNA bases are often promptly identified, removed, and replaced through a step-wise process called base excision and repair (BER), a frontline defense system that prevents genomic instability (7). In essence, once DNA bases are damaged or modified by whatever mechanisms, they are immediately identified and removed by a family of specific DNA glycosylases (9). However, the removal of such DNA bases subsequently generates baseless or apurinic/apyrimidinic (A/P) DNA sites in the genome (9). As the presence of A/P sites, especially in substantial numbers, threatens genomic stability and cell survival, such A/P sites must be repaired in a timely manner to prevent cellular demise or mutagenesis. Interestingly, one of the earliest identified molecules that recognize the A/P sites is Apex1, a molecule that possesses both DNA endonuclease activities and redox functions (10). Apex1 preferentially binds the A/P sites and stabilizes the baseless DNA structure in the genome, and at places where A/P sites are located, Apex1 selectively cuts the phosphodiester bonds in the deoxyribose-phosphate backbone via its DNA endonuclease activities, which transiently creates breaks in the DNA strands (11). Apex1 then recruits complex DNA repair molecules and activates diverse base repair pathways to remove the remaining deoxyribose fragments and restore DNA base integrity (12). Specifically, by recruiting DNA polymerases and DNA ligases to the A/P sites (13), Apex1 helps to reinstall brand new nucleotides at the A/P sites using the complementary DNA strand as a template, steps that are critical to the maintenance of the overall genomic integrity (14, 15).

In the immune system, however, the proliferation of activated T cells is extremely robust and dynamic; they are also under enormous oxidative pressure to become effector cells in a timely manner. Thus, the probability of producing large numbers of modified or damaged DNA bases is high in the proliferating T cells. In fact, certain estimates suggest that as many as 400,000 A/P sites can be generated per cell per day following T cell activation (11). Also, considering the presence of multiple DNA damage repair mechanisms (16), the question as to whether the BER pathway plays any roles in activated T cells, especially the role of Apex1 in the induction of cytopathic T effector cells, becomes an interesting and important question. In the present study, we took a multiplicity of approaches to demonstrate that Apex1 is indispensable in the generation of cytopathic T effector cells in models of autoimmune diseases. We provide further evidence that therapeutically targeting Apex1 with certain chemical inhibitors is highly efficacious in blocking immune-mediated diseases.

## Results

### Identification and conditional deletion of Apex1 for the study of T cell fate decisions.

**Figure 1:**
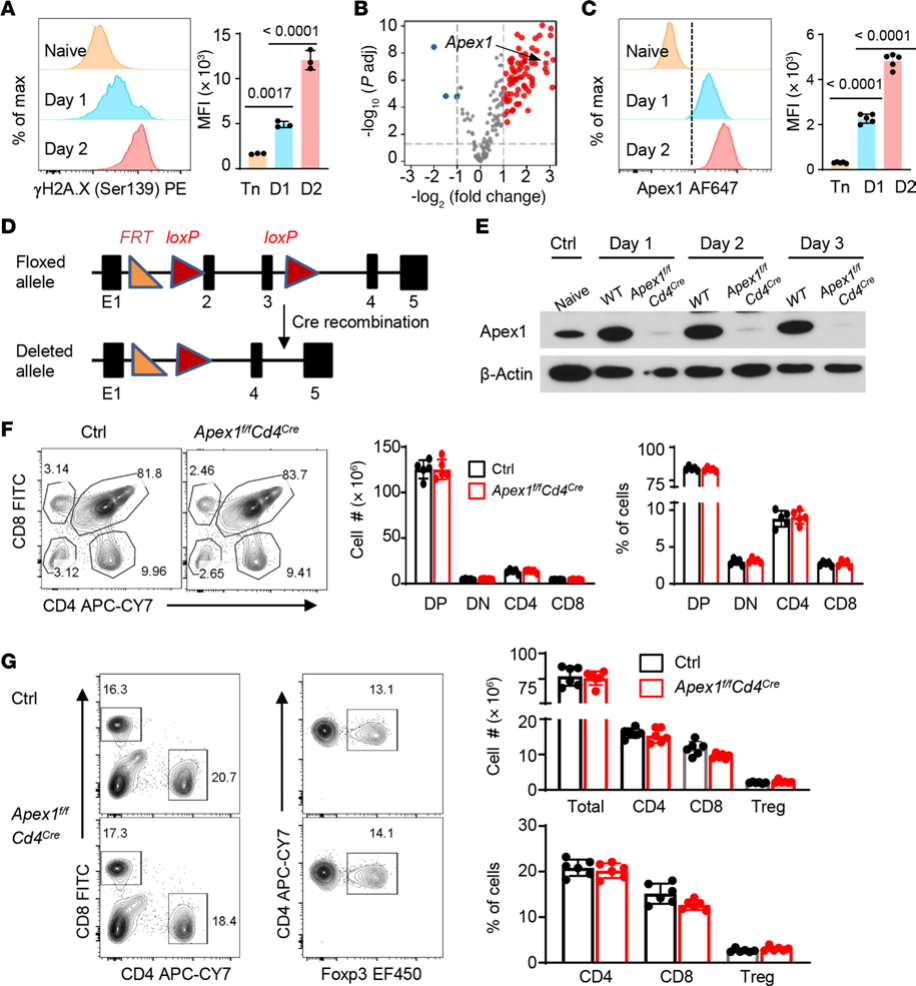
DNA damage repair activities in T cells and the generation of conditionally *Apex1-*deleted mice. (**A**–**C**) WT CD4^+^ T cells were activated with anti-CD3/anti-CD28 for 24 (D1) and 48 (D2) hours in vitro. (**A**) Flow cytometry plots of γH2A.X in both naive (Tn) and activated CD4^+^ T cells (*n* = 3 independent replicates). (**B**) Volcano plot of RNA-seq data illustrating gene expression profiles between naive and activated CD4^+^ T cells 48 hours after activation, highlighting genes involved in DNA replication and base repair pathways (*n* = 3 biologically independent replicates). (**C**) Flow cytometry plots showing Apex1 expression in naive and activated CD4^+^ T cells (*n* = 5 biologically independent replicates). (**D**) Schematic rendering of targeted and deleted *Apex1* allele. The Cre-mediated recombination results in the deletion of exons 2 and 3, which disrupts *Apex1* gene expression in targeted mice. (**E**) Immunoblot analysis comparing Apex1 expression in CD4^+^ T cells from WT B6 and *Apex1^fl/fl^Cd4^Cre^* mice following in vitro activation, with naive WT CD4^+^ T cells serving as an additional control. (**F**) Flow cytometry plots of thymocytes of WT control and *Apex1^fl/fl^Cd4^Cre^* mice, showing 4 different cell subsets based on CD4 and CD8 staining (*n* = 5 mice per group). (**G**) Spleen cells from WT control and *Apex1^fl/fl^Cd4^Cre^* mice were analyzed by flow cytometry, showing comparable CD4^+^, CD8^+^, and Foxp3^+^ subsets (*n* = 6 mice per group). DP, double positive; DN, double negative. For both **F** and **G**, the percentages and absolute cell numbers of the indicated subsets between WT control and *Apex1^fl/fl^Cd4^Cre^* mice are presented in the bar graphs on the right. Data are presented as mean ± SD. Results are pooled from 2 or 3 independent experiments (**A**–**C**, **F**, and **G**). The *P* values were calculated from 1-way ANOVA with Šidák’s post hoc test (**A** and **C**) and 2-tailed, unpaired Student’s *t* test (**F** and **G**).

To determine to what extent the genomic integrity is maintained in productively activated T cells, we firstly assessed the phosphorylation status of the histone variant 2A.X (H2A.X), which serves as a sensitive marker for DNA damage and repair response (17). We FACS isolated naive CD4^+^ T cells from wild-type C57BL/6 (WT B6) mice, activated them with anti-CD3/anti-CD28 in vitro, and stained for the phosphorylated form of H2A.X (i.e., γH2A.X at Ser139). As shown in Figure 1A, we observed that, as compared with the naive T cells, nearly all activated T cells expressed γH2A.X 2 days after activation, and levels of γH2A.X expression, as determined by the mean fluorescence intensity (MFI), were increased by greater than 6-fold over the naive controls (right panel), suggesting a substantial degree of DNA damage repair activities after T cell activation. RNA-seq analysis comparing activated CD4^+^ T cells versus the naive controls identified multiple molecular pathways, especially those involved in DNA replication and DNA base excision repairs, as the highly induced pathways upon T cell activation (Supplemental Figure 1A; supplemental material available online with this article; https://doi.org/10.1172/JCI183671DS1). Interestingly, *Apex1* was identified as one of the highly expressed genes among those pathways (Figure 1B). Flow cytometry analysis using an Apex1-specific monoclonal antibody further confirmed the expression of Apex1 in activated CD4^+^ T cells, either in relative percentage or in MFI (Figure 1C).

To definitively study the role of Apex1 in regulation of T cell activities, we floxed the *Apex1* allele, which involved the insertion of 2 *loxP* sites into the *Apex1* allele flanking the exons 2 and 3 (Figure 1D), and generated *Apex1^fl/fl^* mice. Thus, the Cre-mediated recombination would result in the deletion of exons 2 and 3, thereby disrupting *Apex1* gene expression in targeted mice. We then crossed *Apex1^fl/fl^* mice with *Cd4^Cre^* mice and generated *Apex1^fl/fl^Cd4^Cre^* mice, in which *Apex1* was selectively deleted in all T cells. In immunoblot assays, we confirmed the deletion of Apex1 in anti-CD3/anti-CD28–activated *Apex1^fl/fl^Cd4^Cre^* T cells (Figure 1E). We observed that the adult *Apex1^fl/fl^Cd4^Cre^* mice were healthy, bred normally, and showed no apparent abnormalities as compared with the WT B6 mice (data not shown). Flow cytometry analysis of thymocytes from control (i.e., WT B6, *Apex1^fl/fl^*) and *Apex1^fl/fl^Cd4^Cre^* mice demonstrated similar staining profiles (Figure 1F), with all thymocyte subsets present in a comparable manner, either in relative percentage or the absolute cell numbers (Figure 1F), suggesting that conditional deletion of *Apex1* in T cells unlikely impacts intrathymic T cell development. We also analyzed the spleen and peripheral lymph nodes of age-matched controls and *Apex1^fl/fl^Cd4^Cre^* mice and observed that all the CD4^+^, CD8^+^, and Foxp3^+^ Treg subsets were also comparable in the spleen (Figure 1G), as well as in the peripheral lymph nodes (Supplemental Figure 1B). In a bone marrow chimeric model, in which the WT B6 (CD45.1) and *Apex1^fl/fl^Cd4^Cre^* (CD45.2) bone marrow cells were used to reconstitute the lethally irradiated syngeneic hosts, we observed a certain degree of competitive disadvantage of the *Apex1^fl/fl^Cd4^Cre^* T cells in reconstituting the host spleen and lymph nodes (Supplemental Figure 1C). Collectively, these data suggest that conditional deletion of *Apex1* in T cells does not appear to alter the intrathymic T cell development.

### Conditionally Apex1-deleted mice are protected from autoimmune and allergic tissue damages.

**Figure 2:**
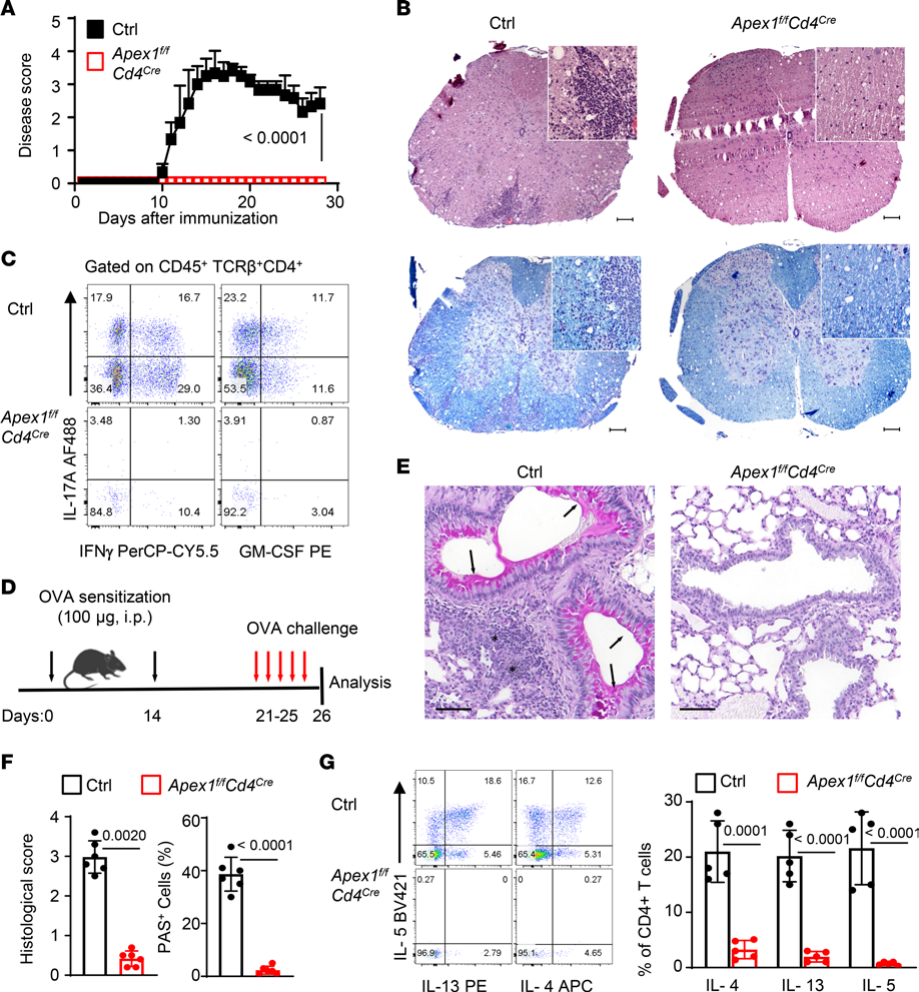
Conditional deletion of *Apex1* protects mice from immune-mediated tissue damage. (**A**) Incidence and clinical scores of EAE in control (*n* = 7) and *Apex1^fl/fl^Cd4^Cre^* mice (*n* = 10). (**B**) Representative H&E (upper) and Luxol fast blue (bottom) staining of spinal cords on day 18 after EAE induction. Scale bars: 100 μm. (**C**) Flow cytometry plots showing IFN-γ^+^, IL-17A^+^, or GM-CSF^+^ cells among CD4^+^ cells from the spinal cords on day 18. (**D**) A cartoon rendering of the induction of allergic airway inflammation in mice. (**E**) Representative periodic acid–Schiff (PAS) staining of lung sections. Arrowheads indicate hyperplasia of mucin-producing cells in airway epithelia and stars indicate tissue inflammation. Scale bars: 100 μm. (**F**) The bar graphs show the lung histological scores (left) and frequency of PAS^+^ mucin-producing cells (right) in indicated mice (*n* = 6 per group). (**G**) Representative flow cytometry plots and bar graphs (*n* = 5 per group) showing frequency of IL-4^+^, IL-13^+^, or IL-5^+^ cells among CD4^+^ T cells from the bronchoalveolar lavage (BAL) of the indicated mice. Data are presented as mean ± SD. The *P* values are from a 2-way ANOVA (**A**) and 2-tailed, unpaired Student’s *t* test (**F** and **G**).

To examine the impact of conditional *Apex1* deletion on the induction of immune-mediated diseases, we initially used an experimental autoimmune encephalomyelitis (EAE) model, in which damage to the CNS is mediated primarily by Th1/Th17 effector cells (18). Briefly, we immunized control and *Apex1^fl/fl^Cd4^Cre^* mice with the myelin oligodendrocyte glycoprotein (MOG) in complete Freund’s adjuvant and monitored for clinical signs of EAE. As shown in Figure 2A, all control mice (both WT B6 and *Apex1^fl/fl^* mice) exhibited prominent clinical signs of EAE, starting on day 10 after immunization and then becoming progressively worse, and by day 18, all the control mice were immobile and moribund. In striking contrast, the MOG-immunized *Apex1^fl/fl^Cd4^Cre^* mice were completely protected, and none of them showed any clinical signs of EAE, which was a surprising outcome. Moreover, tissue histology (H&E) revealed extensive lymphocytic infiltration in the spinal cord of control mice 18 days after MOG immunization, which was associated with substantial tissue damage (Figure 2B). Moreover, Luxol fast blue staining of the spinal cords also revealed extensive demyelination (Figure 2B), pathologies that were consistent with the clinical presentation of EAE. Interestingly, such tissue pathologies were not observed in the immunized *Apex1^fl/fl^Cd4^Cre^* mice (Figure 2B). We also retrieved tissue-infiltrating cells from the spinal cords of MOG-immunized mice and assessed their effector phenotypes by flow cytometry. As shown in Figure 2C, we found that abundant CD4^+^ T cells could be readily recovered from the immunized control mice and a substantial fraction of them (~45%) expressed IFN-γ, IL-17A, and GM-CSF, cytokines that are associated with Th1/Th17 effector cells (19). In contrast, very few CD4^+^ T cells were recovered from the immunized *Apex1^fl/fl^Cd4^Cre^* mice, and most of them retained a quiescent phenotype (Figure 2C and Supplemental Figure 2, A–C). Similarly, in the immunized control mice, the CD8^+^ T cells from the spinal cords also expressed an effector phenotype, but very few CD8^+^ T cells were ever recovered from the immunized *Apex1^fl/fl^Cd4^Cre^* mice (Supplemental Figure 2D). Altogether, these data suggest that in the absence of Apex1, T cells fail to become cytopathic effectors in this model.

To ascertain that the protection afforded by *Apex1* deletion is not unique to EAE, we then examined the role of *Apex1* deletion in a model of allergic lung inflammation, in which the tissue damage relies on the induction of Th2 effector cells (20). We firstly sensitized WT B6 control and *Apex1^fl/fl^Cd4^Cre^* mice with ovalbumin (OVA) in alum, followed by challenging them with aerosolized OVA 21 days later (Figure 2D). As shown in Figure 2E, this protocol consistently induced severe allergic lung inflammation in the control mice, characterized by focal cellular infiltration, prominent airway thickening, and marked proliferation of mucin-producing cells in the airway epithelia, changes that were completely absent in the *Apex1^fl/fl^Cd4^Cre^* mice (Figure 2E). The compiled lung pathologic scores comparing OVA-sensitized and challenged control and *Apex1^fl/fl^Cd4^Cre^* mice demonstrated striking differences (Figure 2F). We also isolated lymphocytes from the airspace of both control and *Apex1^fl/fl^Cd4^Cre^* mice via bronchoalveolar lavage (BAL) and analyzed their cytokine profiles by flow cytometry. As expected, a substantial number of CD4^+^ T cells could be recovered from the control mice, and a substantial fraction of them expressed high levels of IL-4, IL-5, and IL-13 (Figure 2G), cytokines that are typically associated with Th2 cells (21). In contrast, such Th2 cytokines were barely detectable in CD4^+^ T cells from the *Apex1^fl/fl^Cd4^Cre^* mice (Figure 2G). We also detected large numbers of eosinophils in the BAL of control mice, which were completely absent in OVA-sensitized *Apex1^fl/fl^Cd4^Cre^* mice (Supplemental Figure 2E).

We also deleted *Apex1* in MRL/Mp^lpr/lpr^ (*Lpr*) mice, which are known to develop systemic lupus–like disease involving abnormal activation of Tfh cells (22), and found that deletion of *Apex1* (*Lpr*-*Apex1^fl/fl^Cd4^Cre^*) completely inhibited the lupus phenotype (Supplemental Figure 3A). In fact, while the control *Lpr*-*Apex1^fl/fl^* mice progressively developed systemic lupus and died at 24 weeks of age (~75% death rate), all the *Apex1*-deleted *Lpr* mice (*Lpr*-*Apex1^fl/fl^Cd4^Cre^*) survived long term (Supplemental Figure 3A). Additionally, the control *Lpr*-*Apex1^fl/fl^* mice showed proteinuria at 16 weeks of age (Supplemental Figure 3B), which was associated with marked lymphocytic infiltration in the kidney and extensive glomerular damage (Supplemental Figure 3C). Furthermore, analysis using protein chip assays demonstrated increased levels of autoantibodies against a wide spectrum of autoantigens in the control *Lpr*-*Apex1^fl/fl^* mice (Supplemental Figure 3D). Strikingly, all those changes were not observed in the *Lpr*-*Apex1^fl/fl^Cd4^Cre^* mice (Supplemental Figure 3D). Collectively, these findings involving different disease models suggest that Apex1 plays a significant role in the control of cytopathic T cells in vivo.

### Conditional deletion of Apex1 in T cells results in genomic instability and apoptotic death of activated T cells.

**Figure 3:**
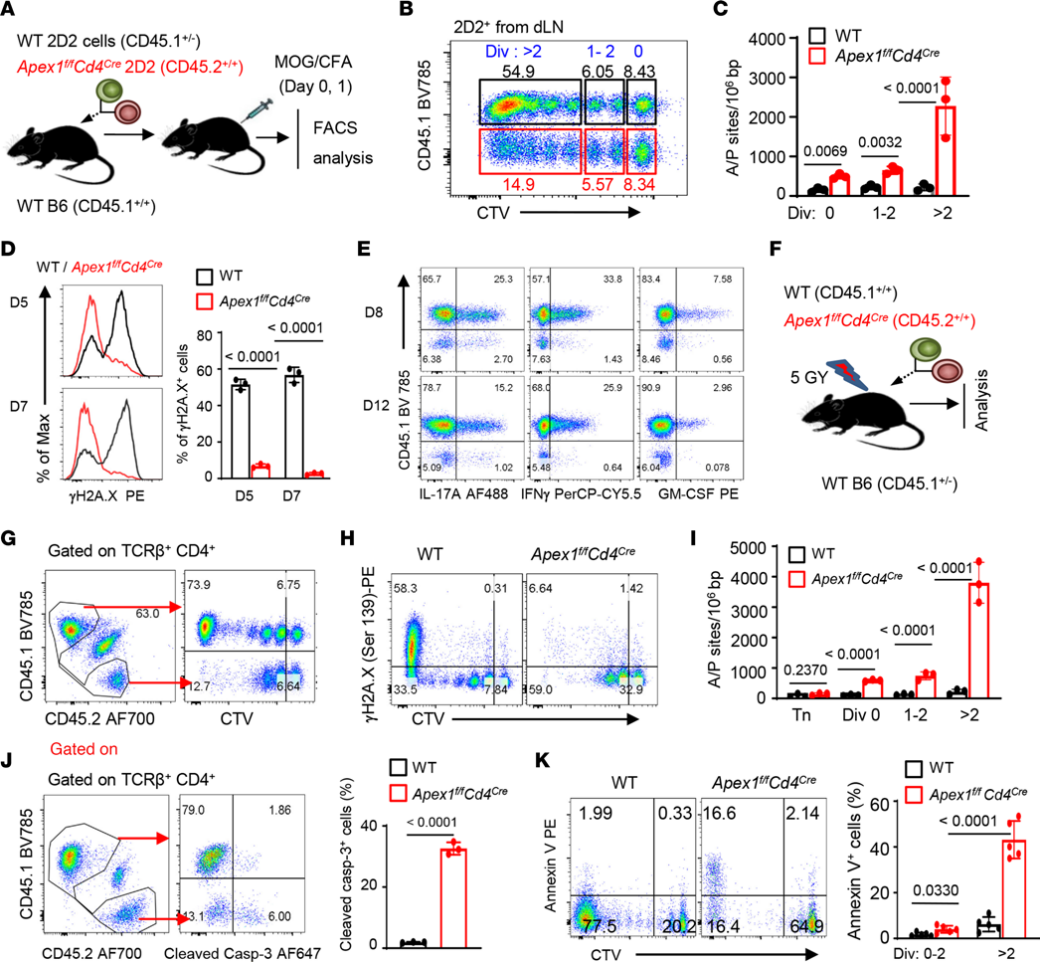
Failure of A/P site repairs results in genomic instability and apoptotic T cell death. (**A**–**E**) WT CD4^+^ 2D2 (CD45.1^+/–^) and *Apex1^fl/fl^Cd4^Cre^* 2D2 (CD45.2^+^) T cells were mixed at a 1:1 ratio (2 million each), labeled with CTV, and co-transferred into CD45.1^+/+^ WT B6 mice 1 day before EAE induction. The transferred 2D2 cells were tracked and analyzed at the indicated time point after EAE induction. (**A**) A cartoon rendering of experimental designs. (**B**) Flow cytometry plot showing frequencies of cell division cycles (0, 1–2, and >2) among the transferred 2D2 cells in the draining lymph nodes (dLNs) 4 days after EAE induction (*n* = 4 mice). (**C**) Bar graphs showing the number of A/P sites in divided 2D2 cells in (**B**) (*n* = 3 mice). (**D**) Representative flow cytometry plots (left) and bar graph (right) showing γH2A.X expression in the transferred 2D2 cells from dLNs on day 5 (D5) and D7 (*n* = 3 mice per group). (**E**) Flow cytometry plots showing the frequencies of effector 2D2 cells from the dLNs on day 8 and day 12 (*n* = 3 mice per group). (**F**–**K**) Analyses of CTV-labeled WT CD45.1^+/+^ and *Apex1^fl/fl^Cd4^Cre^* (CD45.2^+^) CD4^+^ T cells co-transferred into irradiated CD45.1^+/–^ syngeneic B6 mice at a 1:1 ratio (1 million each). (**F**) A cartoon rendering of experimental designs. (**G** and **H**) Representative flow cytometry plots showing cell divisions in vivo (**G**) and γH2A.X expression (**H**) of CTV-labeled CD4^+^ cells in the lymph nodes on day 9 (*n* = 3 mice). (**I**) Bar graphs showing the number of A/P sites among divided CD4^+^ cells on day 5 (*n* = 3 mice). (**J**) Flow cytometry plots showing the frequencies of transferred CD4^+^ T cells and their expression cleaved caspase-3 on day 5 (*n* = 3 mice). (**K**) Flow cytometry plots and bar graphs showing annexin V expression (*n* = 5 mice) among divided CD4^+^ cells on day 5. Data are presented as mean ± SD. The *P* values are from a 2-tailed, paired (**C**, **D**, **J**, and **H**) and unpaired (**I**) Student’s *t* test.

To determine the mechanisms by which *Apex1* deletion impairs T effector cells, we again used the EAE model, in which the WT 2D2 (CD45.1^+/–^) and *Apex1*-deleted 2D2 cells (CD45.2^+^) were adoptively transferred into WT B6 hosts as a reporter cell population (Figure 3A). As the 2D2 cells (CD4^+^) carry a transgenic T cell receptor (TCR) that recognizes the MOG_35–55_ peptide, they can be tracked by staining the transgenic TCR (23), thus allowing real-time in vivo tracking of 2D2 cells for detailed analyses. Additionally, the 2D2 cells could also be labeled with the tracking dye CellTrace Violet (CTV) before adoptive cell transfer, such that their proliferation profiles in vivo could be precisely assessed in the same B6 hosts with or without MOG immunization (Figure 3A). As shown in Figure 3B, WT 2D2 cells proliferated vigorously in vivo in the B6 hosts, and up to 7 cell divisions could be clearly observed 4 days after MOG immunization. Of note, the 2D2 cells that had divided more than 4 times became a dominant cell population at this time (Figure 3B). The *Apex1*-deleted 2D2 cells, however, exhibited much impaired proliferation, especially after 4 cell divisions (Figure 3B). Interestingly, detailed assessments of A/P sites in the genome revealed that the WT 2D2 cells had barely detectable A/P sites in the genome regardless of cell divisions or the number of cell divisions, suggesting a robust A/P site repair mechanism exists in WT 2D2 cells. In stark contrast, the *Apex1*-deleted 2D2 cells displayed a remarkable accumulation of A/P sites, especially in cells that had divided more than 2 times, and as many as 3,000 A/P sites per million DNA base pairs were readily detected in *Apex1*-deleted 2D2 cells (Figure 3C). Moreover, the number of A/P sites in 2D2 cells was inversely associated with DNA damage repair activities, as demonstrated by the γH2A.X staining (Figure 3D). As compared with WT 2D2 cells, the *Apex1*-deleted 2D2 cells recovered from the same hosts failed to show significant γH2A.X staining (Figure 3D). Importantly, WT 2D2 cells in the B6 hosts consistently expressed high levels of IL-17A, IFN-γ, and GM-CSF following MOG immunization (days 8 and 12), confirming their differentiation into T effector cells, whereas the *Apex1*-deleted 2D2 cells showed minimal levels of such effector cytokines (Figure 3E). Similarly, WT 2D2 cells from the CNS of MOG-immunized B6 hosts expressed copious amounts of IL-17A, IFN-γ, and GM-CSF, whereas the *Apex1*-deleted 2D2 cells completely failed to do so (Supplemental Figure 2F).

In a different set of experiments, we co-transferred CTV-labeled WT (CD45.1) and *Apex1*-deleted CD4^+^ T cells (CD45.2) into lethally irradiated syngeneic B6 hosts (Figure 3F), in which the transferred T cells are known to undergo homeostatic proliferation (24). As shown in Figure 3G, the WT CD4^+^ T cells proliferated vigorously in vivo 9 days after adoptive cell transfer, and T cells that had divided 7 times became a dominant cell population. In contrast, the *Apex1*-deleted T cells showed minimal cell divisions. While the highly proliferating WT T cells also exhibited extensive DNA damage repair activities, as revealed by γH2A.X staining (Figure 3H), such DNA repair activities were not observed in the *Apex1*-deleted T cells (Figure 3H). Moreover, WT T cells had barely detectable levels of A/P sites regardless of cell divisions (Figure 3I), confirming that WT T cells possess intrinsic DNA base repair mechanisms. In contrast, the *Apex1*-deleted T cells showed a remarkable accumulation of A/P sites, especially in dividing T cells (Figure 3I). Furthermore, the *Apex1*-deleted T cells showed an increased expression of cleaved caspase-3 (the active form of caspase-3) as compared with the WT T cells (Figure 3J), which was correlated with enhanced annexin V expression by the *Apex1*-deleted T cells (Figure 3K). Together, these data suggest that the vigorously dividing T cells are capable of generating substantial numbers of A/P sites in the genome, and without timely repair of such A/P sites by Apex1, the activated T cells are committed to apoptotic cell death.

### Dependence of Apex1 endonuclease domain for the generation of T effector cells in vivo.

**Figure 4:**
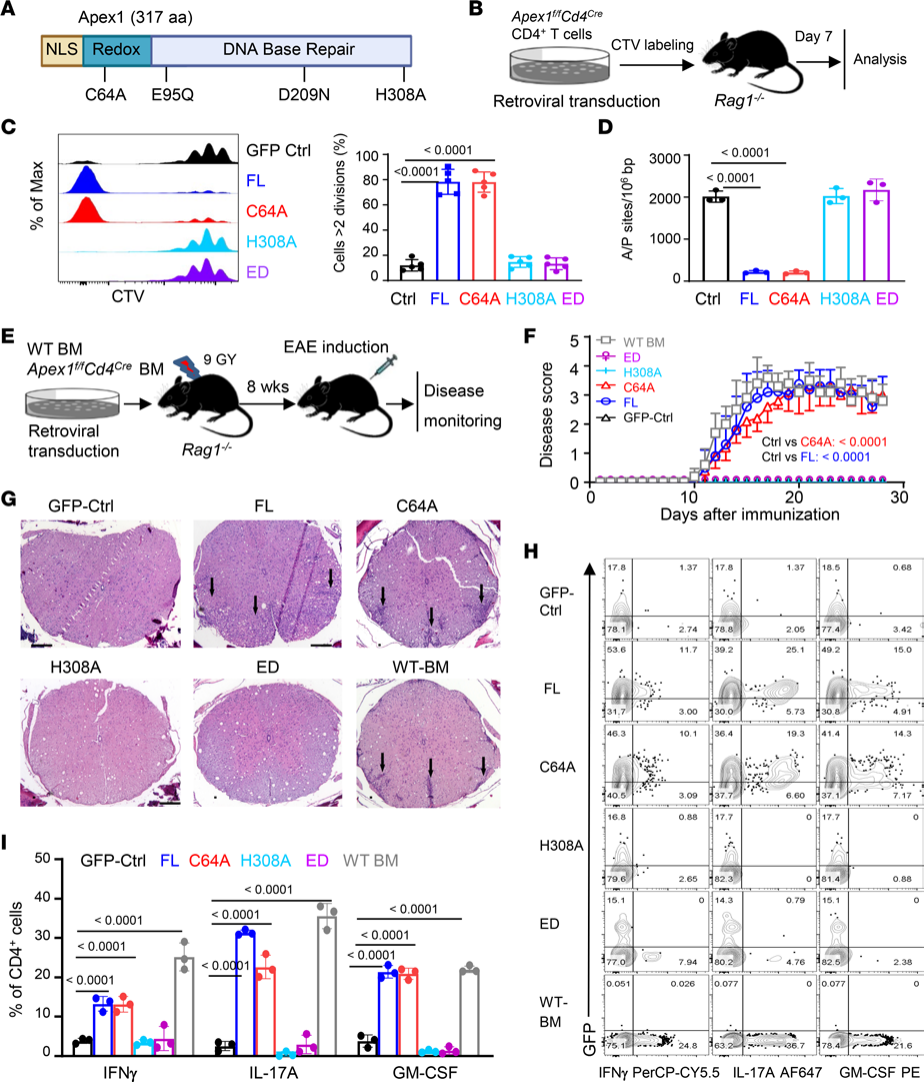
Dependence of Apex1 DNA base repair domain for T cell proliferation and effector functions. (**A**) A diagram illustrating the domain structure of Apex1 and sites of point mutations. (**B**–**D**) *Apex1-*deleted CD4^+^ T cells were activated and spin infected with retroviral vectors containing the full length (FL) or mutants of *Apex1* or GFP-ctrl, labeled with CTV, and then transferred into *Rag1^–/–^* mice for analysis. (**B**) A schematic representation of experimental designs. (**C** and **D**) Representative histograms and bar graphs showing the percentage of cell division (**C**; *n* = 5 mice) and numbers of A/P sites (**D**; *n* = 3 mice) among the CD4^+^GFP^+^ cells on day 7 after transfer. (**E**–**I**) Bone marrow (BM) stem cells from *Apex1^fl/fl^Cd4^Cre^* mice were transduced with retroviral vectors containing various *Apex1* mutants and used to constitute the lethally irradiated *Rag1^–/–^* mice, the reconstituted mice were used as hosts for EAE induction 8 weeks later. (**E**) A diagram showing BM reconstitution and EAE induction. (**F**) Incidence and clinical scores of EAE in BM-reconstituted mice (*n* = 5 mice). (**G**) Tissue pathology of spinal cord sections 22 days after EAE induction (*n* = 3 mice). Arrowheads indicate inflammatory cell infiltration. Scale bars: 100 μm. (**H** and **I**) Flow cytometry plots (**H**) and bar graphs (**I**) showing frequencies of cytokine-producing CD4^+^ T cells in the CNS of host mice on day 22 (*n* = 3 mice). Data are presented as mean ± SD. The *P* values are calculated from 1-way ANOVA with Šidák’s post hoc test (**C**, **D**, and **I**) and 2-way ANOVA with Dunnett’s post hoc test (**F**).

Apex1 is a multidomain and multifunctional molecule, showing both endonuclease (base repair) and redox functions (10, 25). Structurally, the amino acids critically involved in its endonuclease and redox functions have been well documented (Figure 4A) (26, 27). We therefore made a series of *Apex1* mutants, which selectively abolish its base repair versus redox activities, and then tested how such *Apex1* mutants would affect T cell proliferation, A/P site repairs, and effector differentiation in vitro and in vivo (Figure 4A). For this purpose, we transduced CD4^+^ T cells from *Apex1^fl/fl^Cd4^Cre^* mice with a control or retroviral vectors containing various *Apex1* mutants. As the retroviral vectors also contain a GFP reporter, the transduced T cells could be identified by GFP expression. The transduced T cells were allowed to proliferate for 7 days in the *Rag1^–/–^* hosts before all analyses (Figure 4B). As shown in Figure 4C, the *Apex1*-deleted T cells transduced with the full-length *Apex1* proliferated extensively in vivo, and almost all the transduced T cells divided more than 7 times, whereas those transduced with the control vector showed minimal cell divisions. Interestingly*, Apex1*-deleted T cells transduced with the endonuclease-dead *Apex1* mutants (i.e., H308A, E95Q/D209N or ED) failed to show any significant cell divisions, and the highly divided cell population was clearly absent. Interestingly, those transduced with the redox-dead mutant (C64A) proliferated extensively, exhibiting similar proliferation profiles as those transduced with the full-length *Apex1* (Figure 4C). Analysis of A/P sites in the transduced T cells showed that the accumulation of A/P sites was inversely correlated with levels of cell proliferation (Figure 4D), thus demonstrating the importance of the base repair domain of Apex1 in regulating T cell proliferation.

In another set of experiments, we prepared bone marrow stem cells from *Apex1^fl/fl^Cd4^Cre^* mice, transduced them with the *Apex1* mutants, and used them to reconstitute lethally irradiated *Rag1^–/–^* hosts. Here, bone marrow stem cells from WT B6 mice were included as a positive control (Figure 4E). Eight weeks after bone marrow reconstitution, we confirmed that all groups had comparable levels of T cell reconstitution in the periphery (data not shown). We then immunized the reconstituted *Rag1^–/–^* mice with MOG (in complete Freund’s adjuvant) for EAE induction. As shown in Figure 4F, the *Rag1^–/–^* mice reconstituted with WT B6 bone marrow cells developed severe EAE, as did *Rag1^–/–^* mice reconstituted with the full-length *Apex1* and the C64A mutant. In stark contrast, those reconstituted with control vector or the *Apex1* mutants H308A and ED were completely protected, showing no clinical signs of EAE (Figure 4F). Histologically, mice reconstituted with the full-length *Apex1* and the C64A mutant displayed extensive cellular infiltration and spinal cord damage, changes that were not observed in mice reconstituted with *Apex1* mutants H308A and ED (Figure 4G), thus demonstrating an unequivocal role of the DNA base repair domain of Apex1 in promoting cytopathic T effector cell activities in vivo. We also retrieved tissue-infiltrating cells from the spinal cord of reconstituted mice and analyzed them by flow cytometry. As shown in Figure 4, H and I, T cells from mice reconstituted with the full-length *Apex1* and C64A mutant (GFP^+^) expressed high levels IFN-γ, IL-17A, and GM-CSF, while those reconstituted with the H308A and ED mutants failed to do so, thus further demonstrating the importance of the DNA base repair function of Apex1 in cytopathic T effector cell activities.

### Chemical inhibitors suppressing A/P site repair activities of Apex1 inhibit the development of EAE.

**Figure 5:**
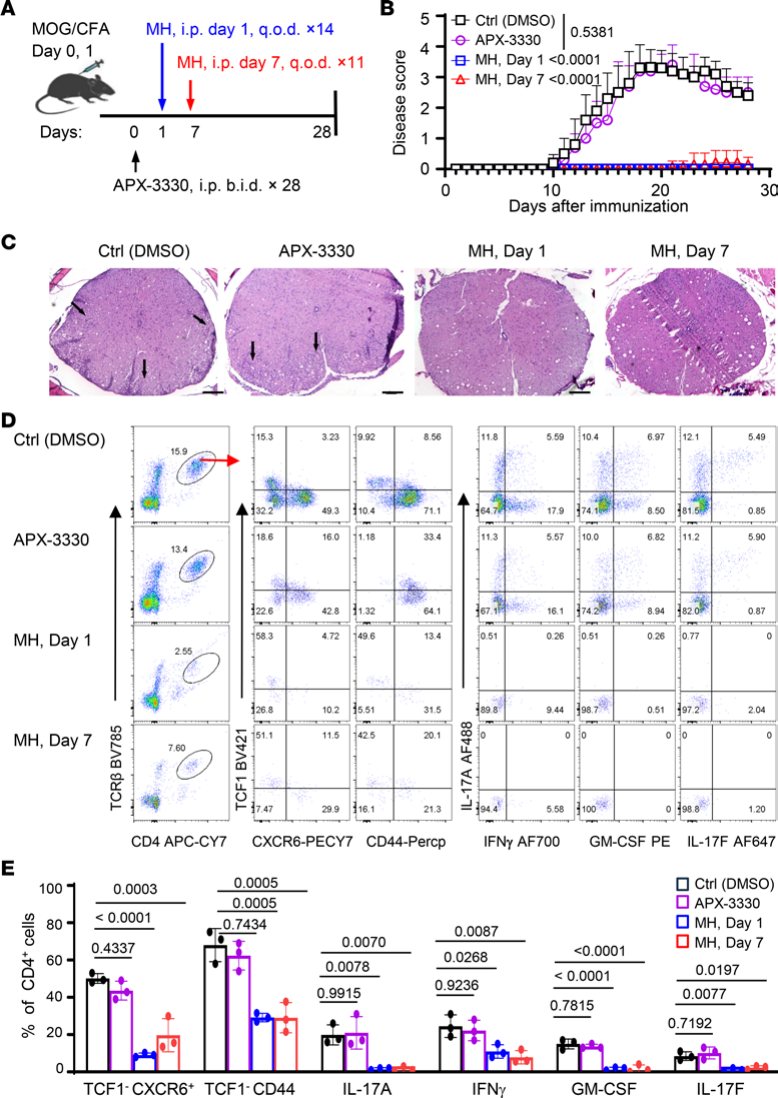
Inhibition of Apex1 base repair activities, but not redox functions, prevents the induction of EAE. (**A**) A diagram showing the workflow for EAE induction and treatment protocols. WT B6 mice were immunized with MOG/complete Freund’s adjuvant on days 0 and 1 and subsequently treated i.p. with vehicle control (*n* = 5), Apex1 inhibitor APX3330 (100 mg/kg, twice daily) (*n* = 5), starting on day 0 for 28 days, or methoxamine hydrochloride (MH, 75 mg/kg, every other day) starting on day 1 for 14 days (*n* = 7) or delayed MH treatment starting on day 7 for 11 days (*n* = 7). (**B**) Incidence and clinical scores of EAE in treated mice. (**C**) Representative tissue pathology of spinal cord sections on day 22 (*n* = 3 mice per group). Arrowheads indicate inflammatory cell infiltrates. Scale bars: 100 μm. (**D** and **E**) Representative flow cytometry plots (**D**) and bar graphs (**E**) showing effector phenotypes and inflammatory cytokine expression by CD4^+^ T cells obtained from CNS of treated mice 22 days after EAE induction (*n* = 3 mice per group). Data are presented as mean ± SD. The *P* values are from 2-way ANOVA with Dunnett’s post hoc test (**B**) and 1-way ANOVA with Šidák’s post hoc test (**E**).

We further examined whether chemical inhibitors of Apex1 would suppress the induction of EAE, focusing specifically on APX3330, a highly selective inhibitor of Apex1 redox functions with proven efficacy in diabetic retinopathy and anticancer activities (28, 29), and methoxyamine hydrochloride (MH), which is known to covalently bind to A/P sites, preventing Apex1 from installing new DNA nucleotides (30). We again immunized WT B6 mice with MOG in complete Freund’s adjuvant, followed by treatment of the immunized mice with vehicle control (DMSO), APX3330 (100 mg/kg, i.p., twice a day for 28 days), or MH (75 mg/kg, i.p., every other day for 14 days); groups of WT B6 mice were also left untreated until 7 days after immunization, allowing T cell priming to occur first, and then treated with MH (75 mg/kg, i.p., every other day for 11 days) (Figure 5A). All the recipient mice were monitored for the development of EAE. As shown in Figure 5B, all control mice developed severe clinical signs of EAE, as did the APX3330-treated mice. Strikingly, all the MH-treated mice were free from EAE, showing no marked clinical signs of the disease. Histologically, mice treated with MH exhibited normal spinal cord structure, with no cellular infiltration and tissue damage, which contrasted sharply with those of control or APX3330-treated mice (Figure 5C). Similarly, flow cytometry analysis of tissue-infiltrating cells from the MOG-immunized mice also revealed strong suppression of Th1/Th17 effector cells by MH treatment when compared with those treated with APX3330 (Figure 5, D and E). These data are consistent with the mutational analysis of Apex1 shown in Figure 4 and further support the importance of the DNA base repair activities of Apex1 in supporting T cell–mediated pathologies.

**Figure 6:**
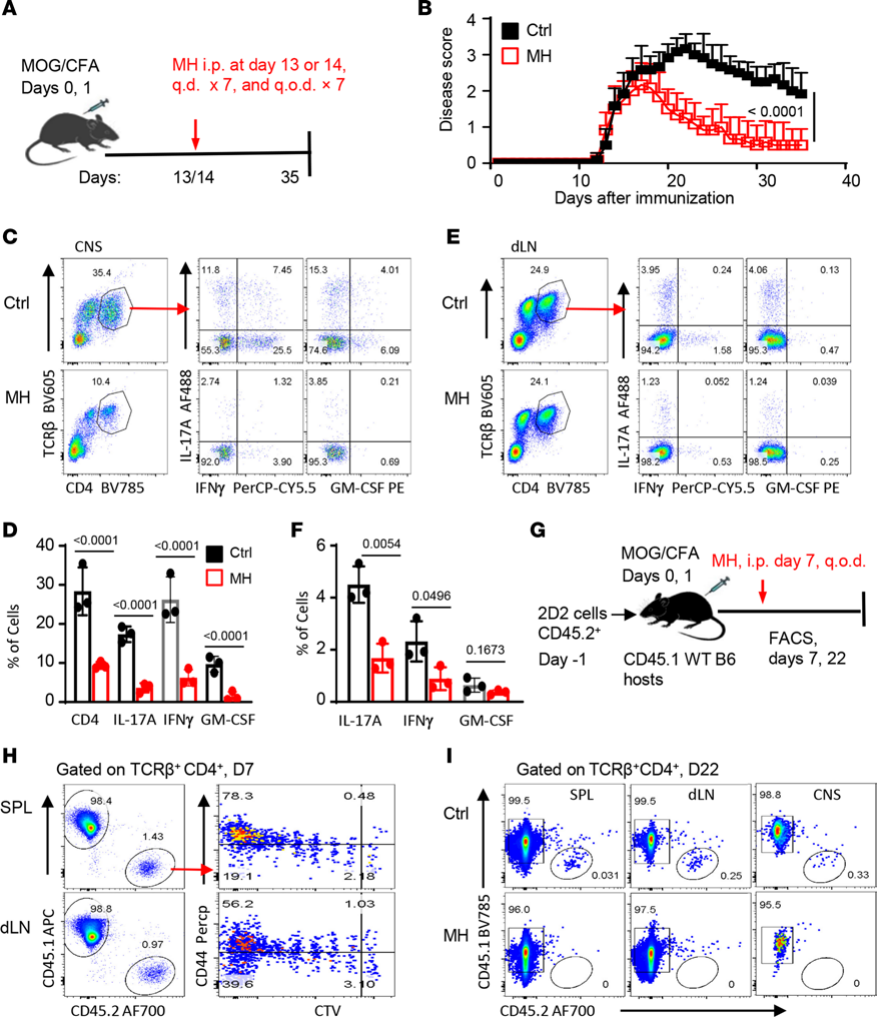
Inhibition of Apex1 base repair activities suppresses EAE progression and promotes disease recovery. (**A**) A diagram outlining EAE induction and MH treatment protocols when the disease was well established. (**B**) Incidence of EAE and clinical scores of control (saline) and MH-treated mice (*n* = 6 in each group). (**C** and **E**) Representative flow cytometry plots showing phenotypes and cytokine expression profiles of CD4^+^ T cells from the CNS (**C**) and draining lymph nodes (dLNs) (**E**) 32 days after EAE induction, and the bar graphs (**D** and **F**) depict the relative percentage of cytokine-producing CD4^+^ T cells in control and MH-treated mice. (**G**–**I**) In vivo tracking of CTV-labeled 2D2 cells (0.25 million per mouse) in MOG-immunized WT B6 mice with or without MH treatment. (**G**) Workflow of EAE induction, 2D2 cell transfer, MH treatment protocol, and analysis. (**H**) Flow cytometry plots showing vigorous proliferation of 2D2 cells in the spleen and dLNs of MOG-immunized mice on day 7 prior to MH treatment. (**I**) Flow plots showing the state of 2D2 cells in the host spleen, dLNs, and CNS on day 22 in control and MH-treated mice (*n* = 3 mice in each group). Data are representative of 1 of 2 independent experiments (**D** and **E**). The *P* values were calculated from 2-way ANOVA (**B**) and 2-tailed, unpaired Student’s *t* test (**C** and **D**).

We performed additional experiments in which the MOG-immunized B6 mice were allowed to develop clinical signs of EAE first and then treated them with the Apex1 inhibitor MH to test whether MH could treat EAE. In this set of experiments, all the host mice exhibited clinical signs of EAE by day 13 and 14 after MOG immunization, showing clinical scores of 1 or 2, time points at which we started treating them with MH (Figure 6A). We observed that, while EAE in the control mice became progressively worse between day 14 and day 25 and reached a disease score of 3 or 4, the MH-treated mice showed a rapid EAE remission and faster recovery (Figure 6B). Flow cytometry analysis showed that CD4^+^ T cells accumulated in substantial numbers in the CNS of control mice, and approximately 30% of them were IFN-γ^+^ T effector cells, and treatment with MH substantially reduced the number as well as their effector activities in the CNS (Figure 6, C and D). In the draining lymph nodes, MH treatment also strongly inhibited the effector T cells (Figure 6, E and F). To further dissect the in vivo effect of MH on T cells, we again transferred CTV-labeled 2D2 cells (CD45.2) into WT B6 mice (CD45.1), followed by immunization of them with MOG and treatment with or without MH (Figure 6G). As shown in Figure 6H, the transferred 2D2 cells initially proliferated extensively in the host spleen and draining lymph nodes by day 7 and failed to survive in the CNS, spleen, and draining lymph nodes by day 22 following MH treatment (Figure 6I). Clearly, the striking effects of delayed MH treatment in suppressing EAE suggest that Apex1 is a promising therapeutic target in treatment of autoimmune diseases.

## Discussion

In the present study, we demonstrated that the A/P DNA endonuclease Apex1 acts as a gatekeeper for the induction of cytopathic T effector cells and highlighted the importance of A/P site repair activities, in which Apex1 plays a central role, in multiple autoimmune disease models and allergic inflammatory tissue damage. In various models and experimental conditions, we provided strong evidence that substantial numbers of A/P sites can be readily generated in the genome of T cells once they are productively activated and vigorously proliferating (not resting cells), in which Apex1 plays a non-redundant role in timely repair of such A/P sites to allow T effector cells to develop. This notion is supported by our findings that either conditional deletion of *Apex1* in T cells, point mutations of its endonuclease domain, or chemical inhibitors of its base repair activities consistently resulted in the accumulation of substantial numbers of A/P sites in T cells, especially in the highly proliferating T cells, followed by their commitment to apoptotic cell death. Thus, in models of autoimmune EAE, allergic tissue inflammation, and systemic lupus, in which diverse Th subsets are intimately involved (19), targeting the Apex1 molecule demonstrated a remarkable efficacy in suppressing the development of such diseases. It should be noted that our current study was focused exclusively on T cells; whether Apex1 also impacts other immune cell types, i.e., myeloid cells and B cells, especially in the in vivo models, and how Apex1 interacts with other base repair pathways (e.g., Apex2) in maintaining the genomic stability, warrant further investigation.

Our finding that the A/P sites serve as a focal point of control of cytopathic T effector cells is highly interesting and potentially important, especially when considering the presence of multiple DNA damage repair pathways in T cells (2, 16). Apparently, the generation of A/P sites, which can be substantial in activated T cells, constitutes a key checkpoint of T cell fate decisions, as their timely repairs allow cell survival and further effector differentiation, whereas their lack of repair results in the activation of cell death programs that promote apoptotic cell death of the activated T cells. The fact that Apex1 regulates this bifurcation makes Apex1 a promising therapeutic target in multiple autoimmune disease settings. However, it should be noted that the precise mechanisms that lead to the accumulation of A/P sites in the genome, how Apex1 itself is regulated in activated T cells, and how the accumulation of A/P sites is mechanistically linked to the T cell death program, especially the activation of apoptotic caspases, remain to be determined. Additionally, the exact mechanisms that endow Apex1 with the exceptional precision in identifying and repairing A/P sites, especially when considering the vast number of DNA bases in the genome (~3 billion base pairs), also remain to be clarified. Nevertheless, our studies provide important insights into the intricate steps involved in maintaining the DNA base integrity in cytopathic T effector cells, knowledge that can be exploited for therapeutic purposes in the clinic.

## Methods

### Sex as a biological variable.

Female mice at 11 weeks of age were used for the EAE experiments, unless otherwise indicated, because of their susceptibility to the induction of EAE and the early onset of the disease. But findings are expected to be of relevance to both sexes. Both male and female mice at 7–10 weeks of age were used for the rest of the experiments.

### Animals.

C57BL/6 (WT B6, CD45.2, stock 000664), B6.CD45.1 (stock 002014), *Rag1^–/–^* (stock 002216), *Cd4^Cre^* (stock 022071), MRL/Mpl^lpr/lpr^ (*Lpr*, stock 000485), and 2D2 TCR-transgenic mice (stock 006912) were purchased from The Jackson Laboratory. WT CD45.1^+/–^ mice were obtained by crossbreeding WT B6 with B6.CD45.1 mice. *Apex1^fl/fl^* mice were generated by Taconic-Artemis in consultation with our laboratory. Briefly, the mouse *Apex1* locus was firstly cloned from a RPCIB-731 BAC library and subsequently re-cloned into a basic targeting vector, placing an F3 site–flanked puromycin resistance cassette in intron 2 and a thymidine kinase cassette downstream of the 3′-UTR, as well as *loxP* site–flanked exon 3. The targeted vector was confirmed by sequencing and the linearized DNA vector was electroporated into C57BL/6N embryonic stem cells, followed by puromycin selection (1 μg/mL) on day 2 and counter selection with ganciclovir (2 μM) on day 5 after electroporation. The targeted embryonic stem cell clones were isolated on day 8 and the targeted allele was confirmed by Southern blotting assays. Blastocysts were isolated from BALB/c females on day 3.5 postcoitum and 10–15 targeted C57BL/6NTac embryonic stem cells were injected into each blastocyst. After recovery, 6 injected blastocysts were transferred into pseudopregnant females. Chimeric offspring were determined by coat color contribution of embryonic stem cells to the BALB/c host (black/white). The chimeric mice were then bred to C57BL/6 females transgenic for the *Flp* recombinase gene to remove the puromycin resistance cassette in mice carrying the conditional knockout allele (*Apex1^fl/WT^*), which were further crossed to generate *Apex1^fl/fl^* mice. Germline transmission was identified by the presence of black strain C57BL/6N offspring. The *Apex1^fl/fl^* mice were backcrossed to the WT B6 background for over 6 generations before subsequent experiments. *Apex1^fl/fl^* mice were further crossed with *Cd4^Cre^* to generate *Apex1^fl/fl^Cd4*^Cre^ mice for all studies.

WT B6, *Apex1^fl/fl^*, or *Cd4^Cre^* mice were used as controls for *Apex1^fl/fl^Cd4^Cre^* mice, and no differences were observed in our investigated phenotypes among those controls. *Apex1^fl/fl^Cd4^Cre^* mice were also crossed with the lupus-prone *Lpr* mice (Jackson Lab). A controlled F2 intercross between heterozygous littermates was used to generate tissue-specific *Apex1*-deficient lupus-prone mice homozygous for deficiency in *Fas* and *Apex1*. The littermates with or without the *Cre* recombinase were analyzed at around 16 weeks of age.

All animals were housed under germ-free conditions at the Houston Methodist Research Institute animal facility under a 12-hour light/dark cycle at temperatures of 20°C–26.1°C and 30%–70% humidity.

### Antibodies, flow cytometry, and cell sorting.

Single-cell suspensions from spleen or lymph node were prepared by physical disruption of such tissues followed by recovering the cells, and cells from bronchoalveolar space and the CNS were processed as previously described (31, 32). Red blood cells were lysed with ACK buffer (A10492-01, Gibco). The viability dye, Zombie Aqua (423102, BioLegend), was used to identify dead cells, and an anti-FcγRII/III antibody (156604, BioLegend) was used to block nonspecific antibody binding. After washing, cells were incubated with mixtures of fluorescently labeled cell surface antibodies for 20 minutes at 4°C. For intracellular cytokine staining, cells were restimulated for 4 hours with phorbol 12-myristate 13-acetate (50 ng/mL) and ionomycin (550 ng/mL; both Sigma-Aldrich) in the presence of GolgiStop (BD Biosciences). Cells were then fixed and permeabilized using a Foxp3 staining buffer set (00-5523-00, eBiosciences) or Cytofix/Cytoperm solution (554714, BD Biosciences), followed by staining with intracellular antibodies according to the manufacturers’ instructions.

Antibodies from BioLegend included FITC–anti-CD8a (53-6.7, 100706, 1:200), BV785–anti-CD8a (53-6.7, 100750, 1:200), APC/Cyanine7–anti-CD4 (GK1.5,100414,1:300), BV785–anti-CD4 (GK1.5, 100453, 1:200), Alexa Fluor 700–anti-CD45 (30-F11, 103128, 1:400), PE–anti-γH2A.X (Ser139) (2F3, 613412,1:20), Alexa Fluor 488–anti–IL-17A (TC11-18H10.1, 506910,1:100), Alexa Fluor 647–anti–IL-17A (TC11-18H10.1, 506912, 1:200), Alexa Fluor 700–anti–IFN-γ (XMG1.2, 505824,1:200), PerCP/Cyanine5.5–anti–IFN-γ (XMG1.2, 505822, 1:200), PE–anti–GM-CSF (MP1-22E9, 505404,1:200), Brilliant Violet 605–anti-TCRβ (H57-597, 109241, 1:200), APC–anti–IL-4 (11B11, 504106, 1:200), Brilliant Violet 421–anti–IL-5 (TRFK5, 504311, 1:200), Alexa Fluor 700–anti-CD45.2 (104, 109822, 1:200), Brilliant Violet 785–anti-CD45.1 (A20, 110743, 1:100), APC–anti-CD45.1 (A20, 110714, 1:200), annexin V–PE (640947; 1:10), PE/Cyanine7–anti-CXCR6 (SA051D1, 151119, 1:200), PerCP–anti-CD44 (IM7, 103035, 1:300), Alexa Fluor 647–anti–IL-17F (8F5.1A9, 517104,1:200), FITC–anti-TCRvβ11 (KT11, 125905, 1:200), PE–anti-Sca1 (D7, 108108, 1:100), and PE–anti–c-Kit (2B8, 105808, 1:100). eFluor 450–anti-Foxp3 (FJK-16s, 48-5773-82, 1:200) and PE–anti–IL-13 (eBio13A, 12-7133-82, 1:200) were from eBioscience. Alexa Fluor 647–anti-TCF1 (S33-966, 566693, 1:100), BV786–anti–active caspase-3 (C92-605.rMAb, 570333,1:100), and Alexa Fluor 647–anti–active caspase-3 (C92-605.rMAb, 570332,1:100) were from BD Biosciences. All samples were acquired using a BD Biosciences LSRFortessa or FACSymphony A5 SE, and data were analyzed with FlowJo v10 software (32). All cell sorting was performed on a FACSAria II (BD Biosciences) (31).

### Activation of CD4^+^ T cells in vitro.

FACS-isolated naive CD4^+^ T cells were stimulated with plate-coated anti-CD3e (5 μg/mL; 2C11; BioLegend) plus soluble anti-CD28 (1 μg/mL; 37.51; BioLegend) at a density of 5 × 10^5^ cells per milliliter. CD4^+^ T cells cultured for 1–3 days under nonpolarizing conditions were subsequently collected and assessed by flow cytometry, immunoblot, and RNA-seq (33, 34).

### A/P site assay.

Genomic DNA extracted from FACS-isolated CD4^+^ cells was prepared using a Genomic DNA Mini Kit (K1820-01, Invitrogen). The measurement of A/P sites was performed using the OxiSelect Oxidative DNA Damage Quantitation Kit (STA-324, Cell Biolabs Inc), according to the manufacturer’s protocols. The aldehyde-reactive probe DNA standards were employed to quantify the number of genomic A/P sites and presented as number of A/P sites per million DNA base pairs (35).

### Cell isolation and retroviral transduction.

The cDNA fragments encoding mouse *Apex1* (full length or *Apex1* mutants) were amplified by PCR and further cloned into the pMYs-IRES-EGFP retroviral vector (RTV-021, Cell Biolabs Inc). Retroviral particles were prepared by transfection of these vectors into packaging Plat-E cells (RV-101, Cell Biolabs Inc) according to the manufacturer’s recommendations. For the transduction of T cells, Dynabeads-purified (11415D, Invitrogen) CD4^+^ T cells were initially activated for 18 hours with anti-CD3e (1.5 μg/mL) plus APC, followed by incubation with freshly prepared retroviral particles by centrifugation for 2 hours at 780*g* and 32°C in the presence of 10 μg/mL polybrene (TR-1003, Sigma-Aldrich). After centrifugation, cells were further cultured for 4 hours at 32°C, followed by culture of the transduced T cells in complete RPM1640 medium at 37°C (32). One day after infection, CD4^+^ T cell were harvested and labeled with CTV and transferred into *Rag1^–/–^* mice for functional studies.

For enrichment of mouse bone marrow stem cells, the EasySep Mouse Hematopoietic Progenitor Cell Isolation Kit (19856, StemCell Technologies) was used, and Sca1^+^ and/or c-Kit^+^ cells were further enriched by positive selection using PE–anti-Sca1 plus PE–anti–c-Kit and anti-PE microbeads (130-048-801, Miltenyi Biotec). Enriched bone marrow stem cells were activated overnight with cytokines IL-3 (10 ng/mL), IL-6 (50 ng/mL), and SCF (100 ng/mL) and then transduced with freshly prepared retroviral particles following the above-mentioned infection protocol. To enhance the transduction efficiency, a second round of transduction was performed on the third day, and bone marrow stem cells were then transferred into 9 Gy–irradiated recipient mice after another 24 hours of culture for further analysis.

### Bone marrow chimeras and adoptive T cell transfers.

For tracing 2D2 TCR-transgenic CD4^+^ T cells in the EAE model, Dynabeads-purified WT CD45.1^+/–^ 2D2 and *Apex1^fl/fl^Cd4^Cre^* (CD45.2^+^) 2D2 CD4^+^ T cells were mixed in a 1:1 ratio (2 million each), labeled with CTV (C34557, Invirogen), and co-transferred into CD45.1^+/+^ WT B6 mice. Subsequently, EAE induction was initiated, and FACS analysis was conducted at various time points for tracking purposes. To study T cell homeostasis, WT CD45.1^+/+^ and *Apex1^fl/fl^Cd4^Cre^* (CD45.2^+^) CD4^+^ T cells were mixed in a 1:1 ratio (1 million each), labeled with CTV, and adoptively co-transferred into 5 Gy–irradiated CD45.1^+/–^ WT B6.

Mixed bone marrow chimera mice were established by transferring 1 million virus-infected *Apex1^fl/fl^Cd4^Cre^* or WT bone marrow stem cells into 9 Gy–irradiated *Rag1^–/–^* recipient mice (36). After an 8-week reconstitution period, chimeric mice were then induced with EAE. In T cell transfers, bone marrow chimeras, both male and female mice were used with sex-matched T cell donors and recipients. In another set of experiments, mixed WT CD45.1^+/+^ and *Apex1^fl/fl^Cd4^Cre^* (CD45.2^+^) bone marrow cells (1:1 ratio, 10 million each) were used to reconstitute the lethally irradiated *Rag1^–/–^* mice. Eight weeks later, T cells in the spleen and lymph nodes of the chimeric mice were examined to determine whether *Apex1*-deleted T cells showed a competitive disadvantage compared with their WT counterparts.

### Immunoblot assay.

Protein extracts were separated by SDS-PAGE, transferred onto an Immobilon membrane, and analyzed by immunoblotting with anti-Apex1 (sc-17774, 1:800, Santa Cruz Biotechnology) and anti–β-actin (12262, 1:2000, Cell Signaling Technology). Horseradish peroxidase–linked antibody against mouse IgG (7076, 1:2000, Cell Signaling Technology) was used as secondary antibody. The expression of target molecules was detected by chemiluminescence, as previously reported (37, 38).

### RNA-seq.

For RNA preparation, 2 million resting and 48-hour-activated CD4^+^ T cells were utilized for RNA extraction with the miRNeasy Mini Kit (QIAGEN). PolyA^+^ RNA was subsequently isolated and RNA-seq libraries were prepared and sequenced at BGI Genomics Corporation. After removing low-quality bases from RNAs, the clean data were aligned to the mouse genome (mm10) using HISAT2 v2.0.5 (https://daehwankimlab.github.io/hisat2). Subsequently, the mapped reads from each sample were assembled using StringTie v1.3.3b (https://ccb.jhu.edu/software/stringtie). FeatureCounts v1.5.0-p3 (https://subread.sourceforge.net) was employed to quantify the number of reads mapped to each gene. Differentially expressed genes (DEGs) were identified using a 1.5-fold variance in expression levels, and an adjusted *P* value less than 0.05 served as the cutoff. The adjusted *P* values were calculated using R software DESeq2 (https://bioconductor.org/packages/release/bioc/html/DESeq2.html). Statistical enrichment of DEGs in KEGG pathways was assessed using the clusterProfiler R package (https://www.genome.jp/kegg/) (39).

### Induction of EAE and treatment protocols.

EAE was induced in host mice using the active EAE induction kit (EK-0113, Hooke Labs) following the manufacturer’s instructions. Mice were monitored daily for signs of EAE and disease severity was scored according to previously reported criteria (32). In some experiments, mice were treated with the Apex1 inhibitor APX3330 (S7445, Selleckchem) or MH (89803-5g, Sigma-Aldrich). A concentrated stock of APX3330 was prepared in DMSO at 100 mg/mL and diluted 1:10 in saline, and mice received 100 mg/kg via i.p. injection twice daily, starting on day 0. A concentrated stock of MH was prepared in saline at 200 mg/mL and diluted 1:10 in saline. Mice received 75 mg/kg via i.p. injection every other day, starting on day 1 (MH, day 1), day 7 (MH, day 7), or days 13–14. DMSO was included as a vehicle control for all treatment protocols (i.p. injections). At the peak of disease or at the indicated time points, mice were euthanized for analysis of CNS-infiltrating cells, as we previous reported (32). Briefly, CNS tissue was minced with a sharp razor blade and digested for 20 minutes at 37°C with collagenase D (2.5 mg/mL; Roche Diagnostics) and DNase I (1 mg/mL; Sigma-Aldrich). Mononuclear cells were isolated by passage of the tissue through a cell strainer (70 μm), followed by centrifugation through a Percoll gradient (40% and 70%). Mononuclear cells in the interphase were removed, washed, and resuspended in culture medium, stimulated with PMA and ionomycin in the presence of GolgiStop for 4 hours, and then were used for analysis by intracellular staining. For histopathological analysis, spinal cords from host mice were fixed in 10% formaldehyde, embedded in paraffin, and sectioned into 5-μm slices. Sections were either stained with Luxol fast blue or H&E. All sections were examined using a Nikon Eclipse E600 microscope.

### Allergic lung inflammation model.

Mice were sensitized by administration of 100 μg of OVA (S7951, Sigma-Aldrich) in 100 μL of Inject Alum (77161, Thermo Fisher Scientific) via i.p. injection. The sensitizing OVA solution was given on day 0 and day 14. Subsequently, sensitized mice were challenged with aerosolized OVA (1% in PBS) for 30 minutes each day over a period of 5 consecutive days (days 21, 22, 23, 24, and 25). The OVA aerosol was generated using a Bennett nebulizer at a flow rate of 10 L/min. One day after the final challenge, all the mice were sacrificed for analyses. Specifically, the lungs were dissected, and the trachea was cannulated with a polyethylene tube for BAL. BAL was collected with 1 mL PBS and centrifuged at 500*g* to separate cells from liquid. The total number of BAL cells was determined by trypan blue exclusion, and then differential cell counts were assessed by cytospins and staining with a Hema 3 staining kit (22-122911, Thermo Fisher Scientific). Lung tissue sections were also used for H&E and periodic acid–Schiff staining. Tissue histopathology and mucin-producing cells and cytokine intracellular staining were assessed as we previously reported (40).

### Proteinuria assay and kidney histology.

The Proteinuria strip (Albustix 2191) was purchased from SIEMENS. The mouse urine was collected in the morning and the proteinuria score was measured according to the instructions. Typically, the score rating is 4 (>2000 mg/dL proteinuria), 3 (>300 mg/dL proteinuria), 2 (>100 mg/dL proteinuria), 1 (>30 mg/dL proteinuria), and 0 (<30 mg/dL proteinuria). Kidneys were removed and washed using PBS before being fixed with 4% formaldehyde. The tissue was embedded in paraffin and processed to 5-μm sections before being stained with H&E.

### Autoantibody array.

Autoantibody profiling was measured by using autoantigen microarrays. Briefly, blood samples from control and *Lpr*-*Apex1^fl/fl^Cd4^Cre^* mice were prepared as indicated and IgG or IgM autoantibody reactivities against a panel of 128 autoantigens were measured using an autoantigen microarray platform developed by the University of Texas Southwestern Medical Center (https://microarray.swmed.edu/products/category/protein-array/) and analyzed by Genepix Pro 7.0 software (Molecular Devices). The data are presented in a heatmap format.

### Statistics.

Data are represented as mean ± SD and were analyzed by Prism software (GraphPad). For the analysis of histology scores, the Mann-Whitney test was applied. The Gehan-Breslow-Wilcoxon test was used for survival analysis. A 2-tailed, unpaired Student’s *t* test or ANOVA was used to generate *P* values, as specified in the figure legends. A *P* value of less than 0.05 was considered statistically significant. The sample sizes are provided in the figure legends to indicate independent replicates used for statistical analyses.

### Data availability.

RNA-seq data for this project have been deposited at NCBI’s Gene Expression Omnibus (GEO) under accession number GSE256454. Raw data are provided in the Supporting Data Values file.

### Study approval.

All animal studies were reviewed and approved by the Institutional Committee on Animal Use and Care at Houston Methodist Hospital in Houston Texas in accordance with institutional guidelines.

## Author contributions

XX and YD designed and performed most of the experiments and therefore are designated as co–first authors. XX is listed first for his efforts in generating the figures and drafting the manuscript. SS, XS, and GW helped with some of the key experiments. JX and ZM provided important help in obtaining and breeding mouse colonies. SME, RMG, AAC, and DZ provided helpful insights and discussions. LJM supported lab operations for the entire project. WC helped in manuscript editing and data analyses. ZZ designed the *Apex1* gene targeting strategy and edited the manuscript. ZZ and XCL supervised the project and finalized the manuscript.

## Supplementary Material

Supplemental data

Unedited blot and gel images

Supporting data values

## Version 1 12/31/2024

In-Press Preview

## Version 2 02/17/2025

Electronic publication
