# Supplementary material for: Apex1 safeguards genomic stability to ensure a cytopathic T cell fate in autoimmune disease models
Source: J Clin Invest. 2024 Dec 31;135(4):e183671. doi: 10.1172/JCI183671 (PMC11827838; doi:10.1172/JCI183671)
Supplement: Supplemental data [file jci-135-183671-s007.pdf]

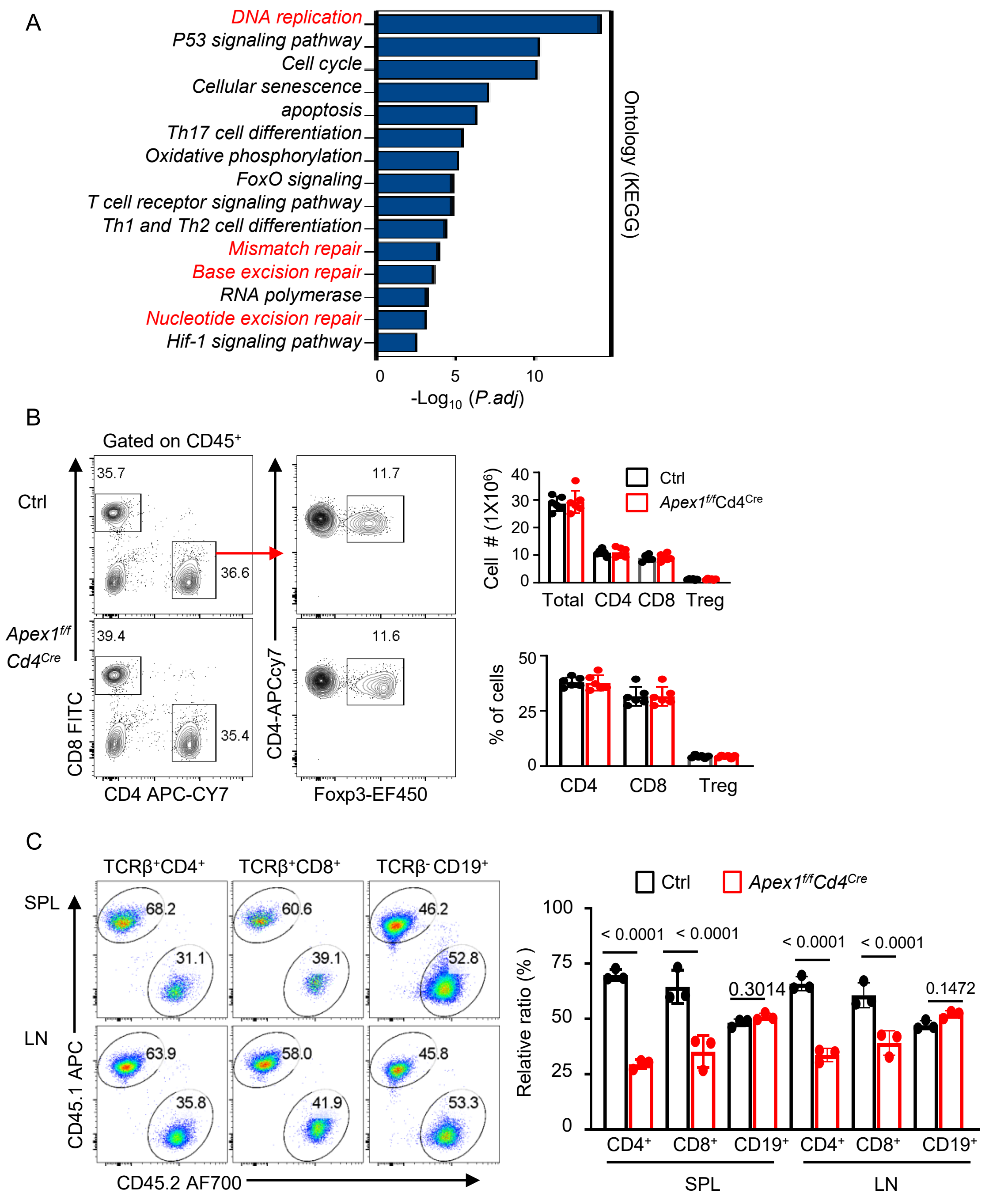

Supplemental Fig 1. See next page for caption.

Supplemental Fig 1. Pathway enrichment analysis, conditional *Apex1* deletion, and T cell subsets in WT *and* $\boldsymbol{Apex1^{f/f}Cd4^{Cre}}$ mice. (A) Gene Set Enrichment Analysis of RNA-seq dataset showing the highly induced top pathways enriched in 48h-activated WT $CD4^{+}$ T cells versus naive $CD4^{+}$ T cells (n=3 biologically independent replicates). (**B)** lymph nodes (LN) of $Apex1^{f/f}Cd4^{Cre}$ and WT control mice were analyzed for the presence of $CD4^{+}$, $CD8^{+}$, and $Foxp3^{+}$ subsets by flow cytometry (left). The percentage and absolute cell number of the indicated subsets were shown in the bar graphs (right) (n=6 mice per group). (**C**) The lethally irradiated (9 Gy) $Rag1^{-/-}$ mice were reconstituted with mixed bone marrow stem cells from WT $CD45.1^{+/+}$ and $Apex1^{f/f}Cd4^{Cre}$ ($CD45.2^{+}$) mice (1:1 ratio, 10 million each), and T cells in the spleen (SPL) and LN in the chimeric mice were examined 8 weeks later and shown. $CD19^{+}$ B cells were included as an internal control and each group included 3 mice. Data are presented as mean ± s.d. p values are calculated by a two-tailed unpaired (B) and paired (C) Student's *t*-test.

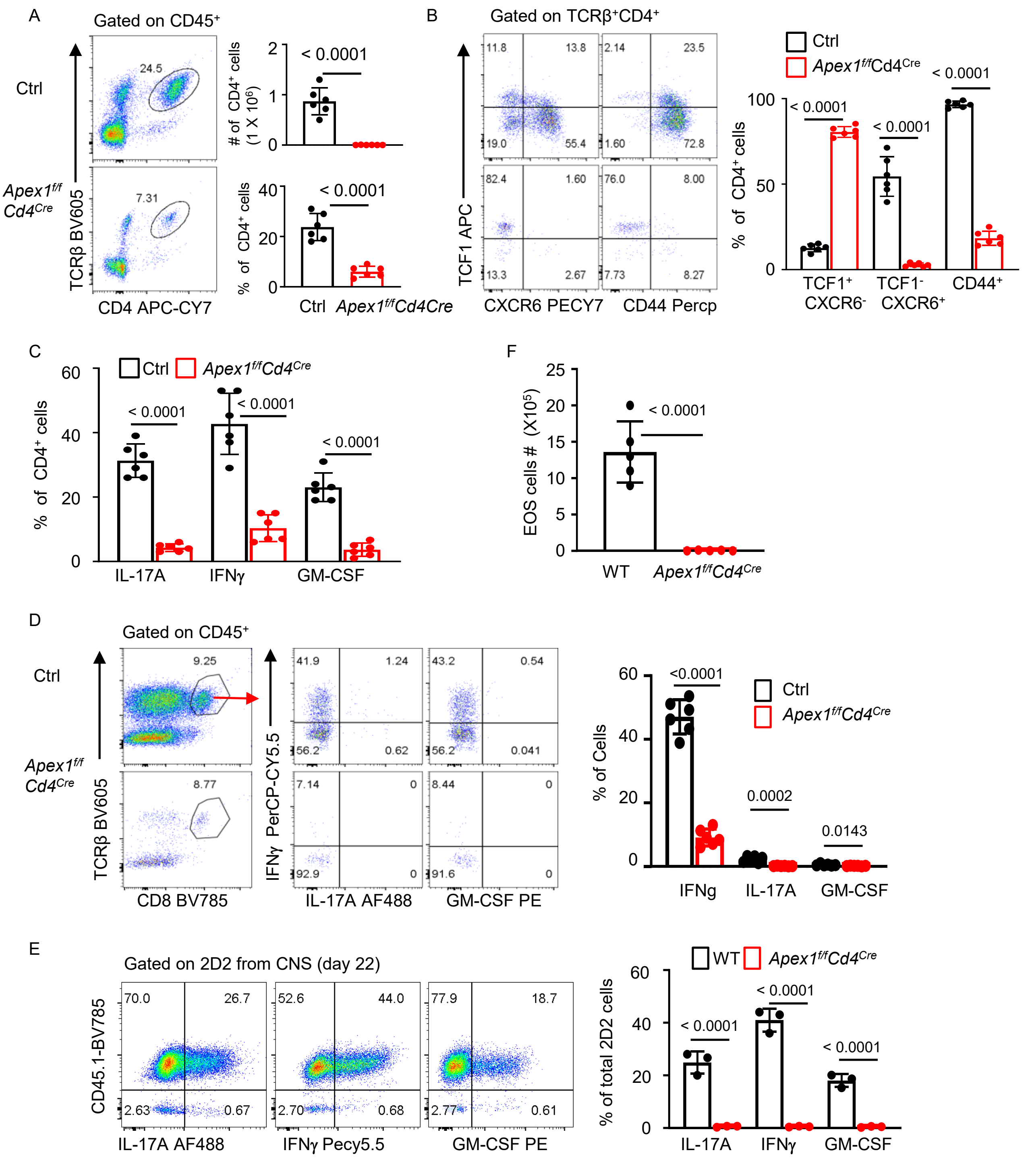

Supplemental Fig 2. See next page for caption.

Supplemental Fig 2. Critical role of Apex1 in the induction of cytopathic T effector cells *in vivo*. (A) Flow cytometry plots (left) and bar graphs (right) showing the percentage and absolute cell number of TCRβ+CD4+ T cells obtained from CNS of EAE mice as stated in Fig. 2C (n=6 mice per group). (**B**) Flow cytometry plots (left) and bar graphs (right) showing the effector phenotype of TCRβ+CD4+ T cells from the EAE mice in supplemental Fig. 1A (n=6 mice per group). (**C**) Bar graph showing the relative percentage of cytokine-producing TCRβ+CD4+ cells from CNS of EAE mice as stated in Fig. 2C (n=6 mice per group). (**D**) Flow cytometry plots showing CD8+ T cells from the spinal cords of MOG-immunized control and Apex1f/fCd4Cre mice, the bar graph on the right is the summary of 6 mice in each group. (**E**) Absolute eosinophil numbers obtained from BAL of OVA-immunized and challenged mice as in Fig. 2G (n=5 mice per group). (**F**) WT 2D2 (CD45.1+/-) and Apex1f/fCd4Cre 2D2 T cells (CD45.2+) were mixed at a 1:1 ratio (2 million each) and co-transferred into CD45.1+/+ control B6 mice 1 day before EAE induction, and expression of inflammatory cytokines on day 22 by the 2D2 cells from CNS were analyzed and shown (n=3 mice). Data are presented as mean ± s.d. and the p values are from a two-tailed unpaired Student's t-test (**A-F**).

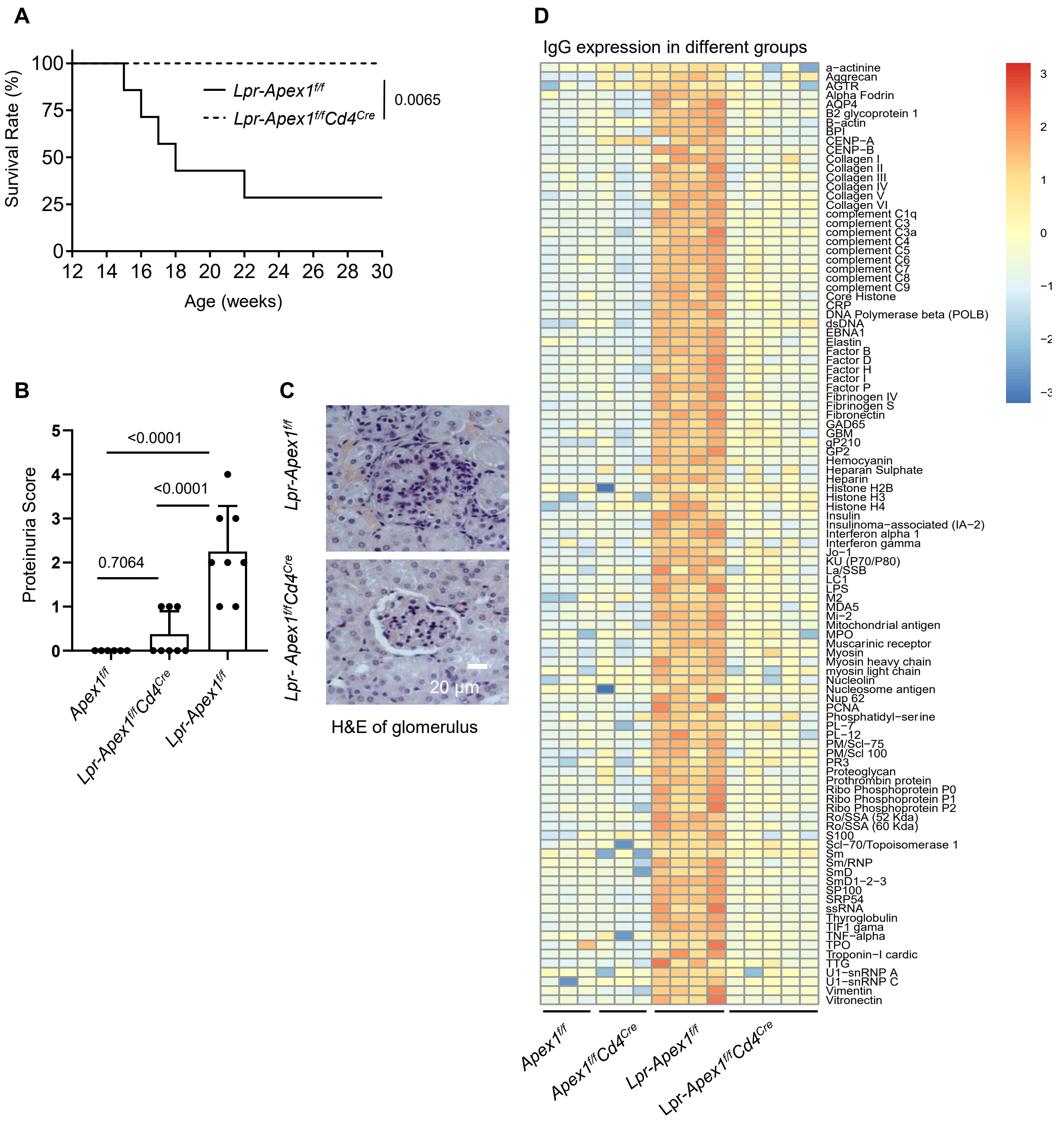

Supplemental Figure 3. Conditional deletion of *Apex1* in T cells in MRL$^{lpr/lpr}$ mice prevents lupus development and reduces autoantibody production. A, Survival curves of Lpr, *Apex1*$^{f/f}$ mice (n=7) and Lpr-*Apex1*$^{f/f}$*Cd4*^{Cre} mice (n=7). **B and C,** bar graphs showing proteinuria measurements (**B**) and representative H&E staining of glomeruli (**C**) of 16 weeks old female littermates of *Apex1*$^{f/f}$ mice (n=6), Lpr-*Apex1*$^{f/f}$*Cd4*^{Cre} mice (n=8) and Lpr-*Apex1*$^{f/f}$ mice (n=8). Scale bar represents 20μm. **D,** Autoantibodies heatmap of IgG against full array of 124 antigens measured in serum of *Apex1*$^{f/f}$ mice (n=3), *Apex1*$^{f/f}$*Cd4*^{Cre} mice (n=3), Lpr-*Apex1*$^{f/f}$ mice (n=4), Lpr-*Apex1*$^{f/f}$*Cd4*^{Cre} mice (n=5), each column represents individual mice in each group. The p value was calculated by Gehan-Breslow-Wilcoxon test for survival analysis (A) and one-way ANOVA (B). Data are representative of three independent experiments (C).
