# Supplementary figures and images for: Apex1 safeguards genomic stability to ensure a cytopathic T cell fate in autoimmune disease models
Source: J Clin Invest. 2024 Dec 31;135(4):e183671. doi: 10.1172/JCI183671 (PMC11827838; doi:10.1172/JCI183671)

Figure 1 E

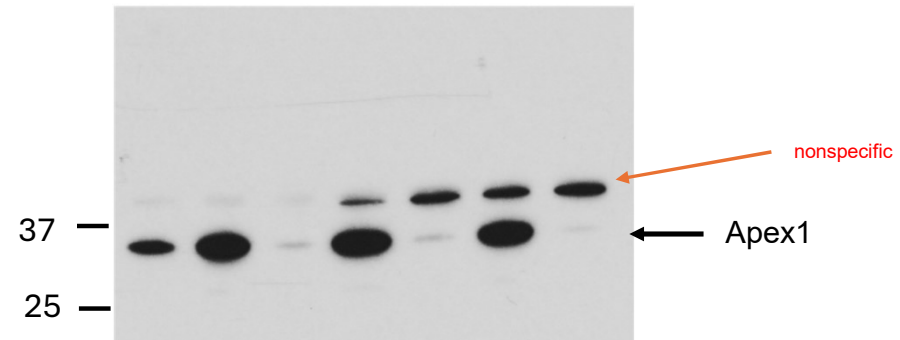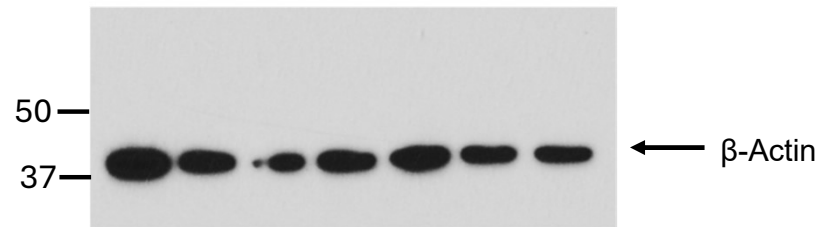

Supplement: Unedited blot and gel images [file jci-135-183671-s008.pdf]
